# Post-activation Potentiation Response of Climbers Performing the Upper Body Power Exercise

**DOI:** 10.3389/fpsyg.2020.00467

**Published:** 2020-03-24

**Authors:** Krzysztof Sas-Nowosielski, Klaudia Kandzia

**Affiliations:** Institute of Sports Sciences, The Jerzy Kukuczka Academy of Physical Education in Katowice, Katowice, Poland

**Keywords:** sport climbing, bouldering, campus board, postactivation potentiation, rate of force development

## Abstract

The purpose of this study was to determine a performance-enhancing effect of post-activation potentiation (PAP) stimulus on climbing-specific upper body power exercises, measured by the IRCRA Power Slap test on a campus board. Two groups of climbers performed the test under one of two conditions: without initial pre-loading (control group) or after 5RM (repetition maximum) pull-ups (PAP group). The test was performed at four time points: at baseline (PRE) and after 4 (POST4), 6 (POST6), and 8 (POST8) minutes of a PAP stimulus (PAP group) or after the same rest period lengths (control group). The results showed that post-baseline slap distances were significantly greater in the experimental group while no change was seen in the control group [repeated measures ANOVA: *F*_(__3_,_42__)_ = 6.26, *p* = 0.001]. *Post hoc* analysis revealed no significant difference between any of the post-baseline trials in both groups. The mean improvement in the first POST4 test in the experimental (PAP) group was +6.5 cm (6.8%). The results of the present study suggest that PAP might be beneficial for acute improvement of upper body power performance in climbers. Therefore we conclude that such stimuli might be advisable for climbers as a part of the warm-up before bouldering competitions and training as well. They might also offer a stronger stimulus for climbers working on power development.

## Introduction

Rock- and sports-climbing continuously increase in popularity; following a recent decision of the International Olympic Committee, sports climbing will enter the program of the 2020 Summer Olympics. The popularity of climbing is also reflected by an increasing number of scientific studies on this activity. While physiological, kinematic, and biomechanical demands of climbing, anthropometric, and physiological characteristics of climbers as well as climbing-related medical problems were frequently studied (Quaine and Martin, 1999; Mermier et al., 2000; Vigouroux et al., 2006; Fuss and Niegl, 2010; Folkl, 2013). However, less is known about sports climbers’ training, including the effects of various exercise protocols, the effectiveness of training modes and methods. Whenever undertaken, such studies have mainly concerned finger strength training on hangboards (Levernier and Laffaye, 2019; López-Rivera and González-Badillo, 2019) and, less often, other aspects of preparation (Philippe et al., 2019). It stands in contradiction to increasing demands of extreme rock-climbing and competition climbing. Climbing, including extreme rock climbing, has long been seen as an activity that requires a high level of muscular strength rather than a great rate of force development (RFD) or power. Nowadays, many top ranked routes demand moves in which a climber has to generate force in an explosive manner. This tendency is even more visible in competitive speed climbing and bouldering. Both disciplines include single movements or sequences of moves that require dynos and jumps, sometimes done in a series, i.e., one after another, bringing to mind rather *le parkour* than climbing that had been practiced a couple of decades ago. As a consequence, biomotor abilities that became of utmost importance for climbers are power and RFD. The former is an amount of force exerted in a unit of time (Zatsiorsky and Kraemer, 2006) while the latter is a measure of how fast an athlete can develop force and is considered a “mechanism behind the expression of power in sport” (Taber et al., 2016, p. 38). Although few researchers have addressed the problem in the context of climbing, the results obtained so far seem important in all climbing disciplines (Fanchini et al., 2013; Levernier and Laffaye, 2019). Not surprisingly, every climber is inclined to include power exercises into their practice regimen.

Among various methods of power development that could be employed into sports climbers’ training is complex training. The term of complex training was introduced by Verkhoskanky, who defined it as “concurrent use of different training means in the same workout, microcycle or mesocycle” (Verkhoshansky and Siff, 1999, p. 365). Considering a single training session, this differentiation mainly refers to selection of exercises which are biomechanically similar, and which should be used in the following sequence: resistance exercise followed by a plyometric, ballistic or speed exercise. The most popular pairs of exercises include squats and jumps, squats and sprints, bench press and clap push-ups, and shoulder presses and overhead medicine ball throws (Seitz and Haff, 2016; Harrison et al., 2019). Such exercise sequences result in a temporal increase in power and force production, thus allowing greater training stimuli and/or enhancing acute performance effect (Docherty and Hodgson, 2007). The physiological rationale for complex training effectiveness is a phenomenon known as post-activation potentiation (PAP), defined as “acute enhancement of muscular performance characteristics as a result of their contractile history” (Tillin and Bishop, 2009, p. 148). Exact nature of PAP is still debatable, and several mechanisms are proposed to explain its effect on performance, e.g., as it act through increasing neural excitability (better motor-unit recruitment and synchronization, decreased presynaptic inhibition), increased amount of Ca^2+^ in the sarcoplasmic reticulum and greater sensitivity of the myofilaments to Ca^2+^, reduction in the sensitivity of Golgi-tendon organs and Renshaw cells thus weakening their inhibitory actions, changes in muscle architecture, and especially a decrease in the pennation angle of muscle fibers with resultant increase of forces that are transferred onto the bones (Scott and Docherty, 2004; Docherty and Hodgson, 2007; Tillin and Bishop, 2009).

Regardless of the true nature of PAP, it seems to induce acute and long term effects on performance in various lower- and upper body activities such as jumps and sprints as well as selected upper-body exercises including bench press throws (Duthie et al., 2002; Docherty and Hodgson, 2007; Liossis et al., 2013; Loturco et al., 2014). To our knowledge only Gołaś et al. (2016) investigated PAP in the upper-body exercises that also involved “pulling” movements – namely Lat pull-downs and dumbbell rows. The main finding of this study was that such exercises might be effective in eliciting PAP in luge athletes. Implementation of these findings into climbing is limited by differences between muscle activity and kinematics characteristic of exercises used by Gołaś et al. (2016) and movements that predominate in climbing (displacement of the body’s center of mass against gravity). One of the most popular forms of climbing-specific power training are campus board exercises (Michailov, 2014), a majority of which are variations of moving up the board from rung to rung (usually referred to as *laddering*), reaching explosively upwards as far as possible with one hand (usually called *reaches* or *touches*) or two hands (usually called *doubles* or *dynos*). Campus exercises are considered an “extraordinary tool for developing explosive strength, improving force gradient, intramuscular and intermuscular coordination” (Michailov, 2014, p. 103) and are executed with the repetitive and/or interval methods. As research data on the effectiveness of PAP on campus boards, and climber’s training in general, are scarce, the purpose of this study was to determine a performance-enhancing effect of PAP stimulus on climbing-specific upper body power exercises.

## Materials and Methods

After providing a written informed consent, a total of 16 climbers (including five females), aged 22–31 (*M* = 27.44, SD = 2.76) were recruited to the study. All were members of two athletic teams (*n* = 10 and *n* = 6) in one of the bouldering gyms in Katowice, south Poland. All participants were advanced climbers practicing from 5 to 15 years, familiarized with campus board exercises. Their climbing performance level was determined based on self-reported best red-point (RP) climbs ranging from 7b+ to 8c in a French grading system or 22–31 in the International Rock Climbing Research Association (IRCRA) Reporting Scale, so they could be classified as advanced-to-elite (Draper et al., 2011). Detailed characteristics of the participants are presented in Table 1. The climbers were randomly assigned to PAP-stimulus condition (experimental group) or to the control group. There were no significant differences in climbing experience [*t*_(__14__)_ = 0.35, *p* = 0.730], the level of advancement [*t*_(__14__)_ = 0.22, *p* = 0.976], body weight [*t*_(__14__)_ = 1.99, *p* = 0.066], height [*t*_(__14__)_ = 1.84, *p* = 0.087] or BMI [*t*_(__14__)_ = 1.16, *p* = 0.266]. The only variable that differentiated both groups was age [*t*_(__14__)_ = −2.17, *p* = 0.048]. Detailed data are presented in Table 1.

**TABLE 1:** Descriptive characteristics of the study participants (mean ± SD).

	**All**	**Experimental**	**Control**
Age (years)	27.4 ± 2.8	26.4 ± 2.7	29.2 ± 1.9
Experience in climbing (years)	8.69 ± 3.03	8.9 ± 3.4	8.3 ± 2.5
Body mass (kg)	66.9 ± 10.7	70.7 ± 10.5	60.7 ± 8.21
Height (cm)	173.9 ± 6.1	175.9 ± 6.0	170.5 ± 5.0
BMI	22.2 ± 2.2	22.7 ± 2.2	22.2 ± 2.2
Climbing level (IRCRA Reporting Scale)	25.1 ± 3.2	25.2 ± 3.2	24.8 ± 3.4

*BMI, body mass index.*

**FIGURE 1:**
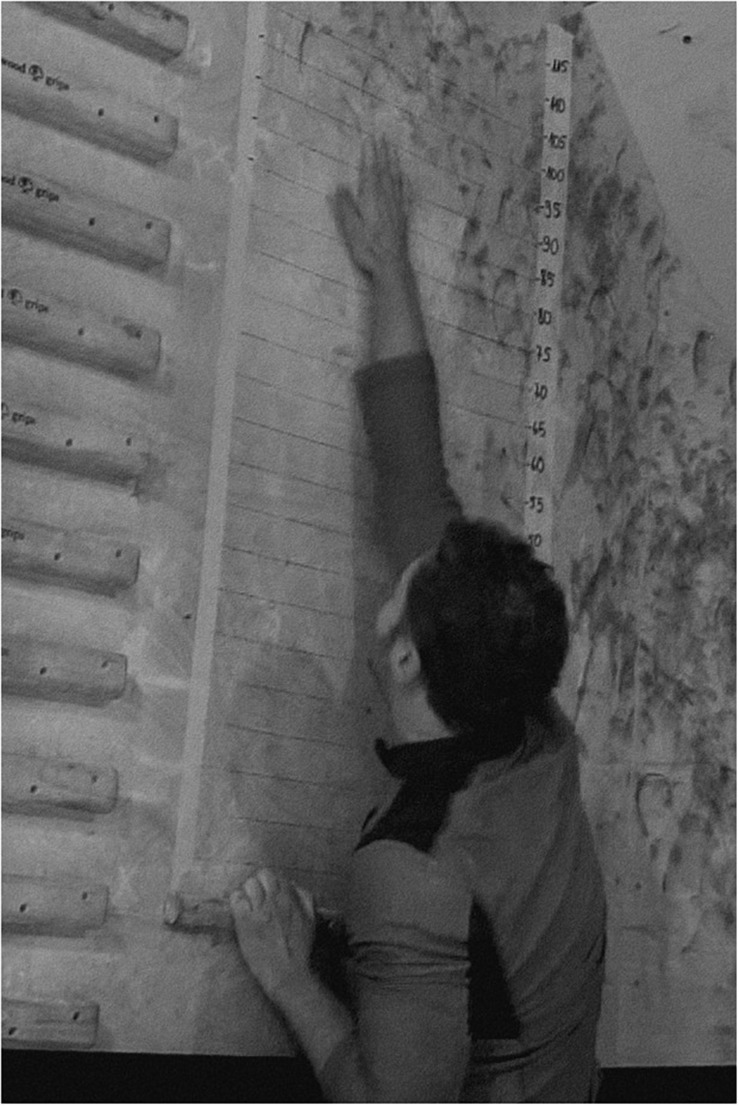
The finishing position of the Slap test.

As all the exercises were previously regularly performed by the participants as a regular part of their training program, no familiarization session was included in the present study. Prior to testing, the participants were instructed to perform a warm-up according to their individual preferences in order to prepare for intense campus exercises. They were not required to do a standardized warm-up protocol as we assumed that, as advanced climbers, they were experienced enough to know how to prepare themselves best for particular climbing efforts. The participants could use wooden and resin hangboards, i.e., Beastmaker 2000 (Beastmaker, United Kingdom) and MocArt (MocArt, Poland), respectively. There was a campus board with two kinds of rungs, a system wall with big sloper-like rungs and wooden hemispheres, boulder walls, the Moon system wall, TRX suspension system, gymnastic rings, a pull-up bar, and a set of dumbbells. After the warm-up, the test of 5RM (repetition maximum) pull-up exercise was performed using a direct assessment method with 2 min rests between trials. Participants performed pull-ups on a Beastmaker 2000 fingerboard using two deep four-finger pockets held with a half-crimp grip and spaced 56 cm apart measured between their outer edges. They were instructed to do the pull-ups starting with their arms fully extended to a position in which the chin reached the level of the holds. The value of the external load corresponding to 5RM ranged from 10 to 45 kg (*M* = 25.40, SD = 10.70). The test session was performed after a one-day break, during the successive training session. IRCRA Power Slap (IRCRA, 2015), chosen as a power test, was performed on a board on which a scale with distances in centimeters was drawn. A 2.5-cm deep rung (Modell 2 by Tripoint, Tripoint, Poland) was placed at the bottom of the board. The rung allowed curling the fingers over its grip (“positive grip”) to minimize the possibility of slipping off the rung during the pulling movement. According to the IRCRA recommendations, the manual climber’s task was to hold on the rung with straight arms and initiate an explosive pull-up and slap as high as possible with one, dominant, arm. The performance was measured by a direct measurement method using the magnesia mark left by the climber’s hand. To ensure greater accuracy and minimize the risk of blurring earlier magnesia traces, each climber was video-recorded (Figure 1).

Participants from the experimental group were instructed to perform one set of 5RM pull- ups with a pre-determined load. Pull-ups were to be done in a row, without stopping. Power Slap test started after a 4-min break (POST4). Break duration was chosen for two reasons. Firstly, according to Lowery et al. (2012), rest periods of 4–8 min are close to optimal (Wilson et al., 2013). Secondly, 4 min is a typical rotation time in bouldering competitions and during establishing the study protocol we had to bear in mind a practical aspect of our research. The test was repeated twice, i.e., after 6th (POST6) and 8th (POST8) minute of the PAP exercise – again to simulate the rotation time in bouldering competitions. Before each trial climbers were allowed to use chalk – the one which they usually use in their climbing.

The study protocol was approved by the Ethics Committee of Biomedical Research at the Academy of Physical Education in Katowice – resolution no 1/2019.

## Data Analysis

Assumptions of normality and homogeneity of variance were tested with the Shapiro–Wilk and Levene’s tests, respectively. The student *t*-test for independent samples was used to compare characteristics of control and experimental groups. Repeated measures ANOVA with Tukey *post hoc* test was used to assess the effects of the PAP exercise (pull-ups) on muscle power output. All statistical analyses were conducted using Statistica 13.3 (Statsoft, Poland) software.

## Results

Descriptive statistics (means, standard deviations, and confidence intervals) of the study results are presented in Table 2.

**TABLE 2:** Comparison of Power Slap results in centimeters (*M*, SD, CI’s) between two groups, PAP and control in PRE and POST conditions.

	**Experimental**				**Control**			
	**Mean**	**SD**	**−95% CI**	**+95% CI**	**Mean**	**SD**	**−95% CI**	**+95% CI**
PRE	94.5	12.1	85.8	103.2	85.8	10.2	75.1	96.5
POST4	101.0	13.5	91.3	110.7	85.0	10.5	74.0	96.0
POST6	100.5	15.9	89.1	111.9	84.2	9.2	74.5	93.8
POST8	100.5	16.2	88.9	112.1	85.0	11.4	73.0	97.0
*post hoc*	PRE–POST4: *p* < 0.001				PRE–POST4: *p* = 0.896			
	PRE–POST6: *p* < 0.001				PRE–POST6: *p* = 0.390			
	PRE–POST8: *p* < 0.001				PRE–POST8: *p* = 0.428			

*POST4, POST6, and POST8 – Power Slap at 4, 6, and 8 min after PAP (for the PAP group) or after resting time of the same duration as the PAP (for the control group).*

The difference in distances obtained by both groups during the PRE trial was not statistically significant [*t*_(__14__)_ = 1.46, *p* = 0.166]. PRE-POST comparisons revealed a significant effect of PAP stimulus on the Power Slap exercise on a campus board, *F*_(__3_,_42__)_ = 6.26, *p* < 0.001, η^2^ = 0.24, non-centrality = 4.45. Compared to the baseline, none of the three successive trials was significantly different in the control group whereas in the experimental group all three tests differed significantly (*p* < 0.001). No significant differences were revealed between POST4, POST6, and POST8 tests in both groups.

The mean improvement in the first POST test in the experimental (PAP) group was +6.5 cm while in the control group a slight decrease of −0.83 cm was observed. It should be noted that each participant in the experimental group improved while the control participants obtained the same distance, except for one climber whose result decreased by approximately 5 cm.

## Discussion

The phenomenon called PAP has drawn attention of sports scientists, athletes, and sports coaches for years. Its essence is “the increase in muscle force and RFD that occurs as a result of previous activation of the muscle, as well as the force and power of evoked high velocity shortening contractions, and the maximum velocity attained by evoked shortening contractions under load” (Lorenz, 2011, p. 235). Although the issue remains controversial, previous studies reported a possible ergogenic effect of PAP on acute performance and chronic conditioning strategy, i.e., the so called complex training (Docherty and Hodgson, 2007; Tillin and Bishop, 2009; Wilson et al., 2013; Helena et al., 2019). Most studies were conducted on pairs of lower body activities like squats and vertical jumps, squats and sprints, loaded sprints and unloaded sprints etc. (Tillin and Bishop, 2009; Dobbs et al., 2019). Fewer studies examined the effects of PAP on the upper body exercises (Ebben, 2002) like bench press and ballistic push-ups (Farup and Sørensen, 2010; Liossis et al., 2013; Seitz and Haff, 2016) where the movement predominantly involved some “pushing” action. For that reason their findings cannot be directly translated into climbing in which “pulling” actions are typical. The only study which, to our knowledge, investigated the effects of PAP on pulling movement patterns was that of Gołaś et al. (2016); the authors determined the impact of Latissimus pull down exercise performed on the Keiser Power Rack at 50% 1RM and dumbbell row at 80% 1RM (three sets of four reps with a 90 s rest interval) on the luge start. It was found that the PAP protocol of dumbbell rows significantly improved power during the Keiser Pull Down following 6 min of recovery and that a very significant correlation existed between the power generated during the Latissmus Pull Down and the luge start. While these findings are in accordance with other studies, it should be pointed out that Lat pull-down is a type of an open kinetic chain manoeuvre (the trunk stabilized, bar pulled toward the chest) that creates different kinematic conditions and different stimuli than in pull-up movements characteristic of climbing (Johnson et al., 2009; Doma et al., 2013). For that reason findings of Gołaś et al. (2016) might not apply to climbers’ training and pre-competition warm-up protocols.

Therefore, the purpose of this survey was to assess acute effects of PAP in a climbing-specific power exercise, performed on one of the most popular training devices, namely the campus board, which is also suggested to be the testing tool in power and power endurance assessment. The power exercise chosen for this study, consisted of pulling up explosively from a hanging position with straight arms, and reaching with one dominant hand as high as climbers could touch. This test is recommended by IRCRA to assess the upper body power as it simulates explosive movements done in climbing. Considering the criterion of biomechanical similarity between complementary exercises (PAP-eliciting resistance exercise and the target explosive exercise), resisted pull-ups on a fingerboard were chosen. It was not without significance that this kind of exercise is among the most frequently performed resistance exercises in climbers’ preparation and is familiar to the most, if not all, advanced climbers. The intensity of the pull-ups was 5RM, which corresponded to ca. 85–87% of 1RM, i.e., the intensity within the range considered as the most effective for eliciting PAP (Carter and Greenwood, 2014). The results showed that loaded pull-up done for 4 min before the “power slap” had a positive effect on the latter, allowing the athlete to reach higher than without such preconditioning. Moreover, this potentiating effects last up to 8 min after the PAP stimulus, which may be an important consideration for climbers taking part in bouldering competitions during which a period for completing the problem, known as a “rotation time” lasts 4 min in the final round. A time course found in this study is comparable to the findings of Nibali et al. (2015), who compared 4, 8, and 12 min intervals in PAP response of jump squats and found that, despite individual variations, the 4-min interval displayed the greatest magnitude and frequency of potentiation. However, at this stage one should refrain from recommending any time interval as optimal.

To the best of our knowledge, this is one the first studies in which the effects of PAP on the climbing-specific upper body power exercises were assessed. Therefore, the comparison between the findings of the present study and other literature reports is hardly possible. The only study for comparison is our previous one (Sas-Nowosielski and Kandzia, 2018), which was however, conducted without a control group and with only one time point after PAP stimulus. Although similar findings had been obtained, not all participants had responded positively to the stimulus (a few climbers showed no improvement). In fact, such variations in response were also observed in other studies involving such exercises like squats and jumps (Duthie et al., 2002; Gourgoulis et al., 2003). Such variable responses to preload stimuli have been attributed to strength and training status of athletes, with stronger individuals usually showing greater PAP response than the weaker ones, or to different proportions of slow- and fast muscle twitch fibers in various individuals (Tillin and Bishop, 2009; Seitz and Haff, 2016; Chen et al., 2017). In the latter case it is suggested that the fast twitch fibers react with greater phosphorylation of regulatory light chains and may therefore be more prone to positive response to PAP stimulus (Tillin and Bishop, 2009). All experimental group climbers in our study responded positively and only differed as to the magnitude of the improvement.

## Limitations

There are a few shortcomings of this study that need to be considered when interpreting its results. Firstly, quite a small number of subjects limits data analysis as the PAP group climbers could not be further divided into subgroups of different strength levels. It has been previously reported that PAP response could be influenced by the level of strength, with stronger individuals exhibiting stronger response. It would be interesting to check whether this also applies to campus board performance. Secondly, the analysis was limited to one parameter, i.e., the distance obtained in the “Slap” test; other parameters, such as power or velocity were not assessed. Finally, after random allocation of the participants into the PAP and control groups, they were measured once, while it would be more informative if cross-over measurements had been performed. While it was a consequence of participants availability, in the future research the cross-over measurement should be also considered.

## Practical Applications

Based on the results of the present study, the use of PAP can be recommended to acutely enhance the upper body power performance of climbers. It is especially important when we consider bouldering contests which tend to feature at least one of the “problems” as highly dynamic. Efficient problem solving largely depends on climbers’ abilities to exert high RFD. While bouldering is, by definition, associated with power, lead climbing is considered an activity to test climbers’ anaerobic and mixed aerobic/anaerobic endurance. However, one should bear in mind that a shift toward a more spectacular, dynamic-style is also seen in lead climbing. As a consequence, the lead climber is forced to perform one or even several jumps and dynos. It can be perceived as a new challenge for lead climbers, who may also benefit from including PAP-eliciting exercises into their pre-competition warm-up protocol.

## Conclusion

Although PAP and its impact on various exercises and activities (vertical jumps, sprints, long jumps, and dynamic push-ups) have been studied for many years, its application to sport climbing performance has received less attention. We believe that the present study is noteworthy for coaches and climbers as it confirms that this phenomenon may find application in acute power performance. Still, more research is needed to determine the best strategy for using PAP in climbing – both regarding its acute and chronic effects. A list of problems that deserve attention may include, for example, long term effects of using PAP in training, delayed potentiation, optimal time and loading protocols of exercise pairs in sports climbers’ training and pre-contest conditioning.

## Data Availability Statement

The datasets generated for this study are available on request to the corresponding author.

## Ethics Statement

The studies involving human participants were reviewed and approved by Bioethical Commission of the Academy of Physical Education in Katowice. The patients/participants provided their written informed consent to participate in this study. Written informed consent was obtained from the minor(s)’ legal guardian/next of kin for the publication of any potentially identifiable images or data included in this article.

## Author Contributions

KS-N and KK contributed conception and design of the study, organized the database, and read and approved the submitted version. KS-N performed the statistical analysis and wrote the first draft of the manuscript.

## Conflict of Interest

The authors declare that the research was conducted in the absence of any commercial or financial relationships that could be construed as a potential conflict of interest.
